# Tumor regression and survival after perioperative MAGIC-style chemotherapy in carcinoma of the stomach and gastroesophageal junction

**DOI:** 10.1186/s12893-015-0054-9

**Published:** 2015-05-22

**Authors:** Fernando Mingol, Javier Gallego, Albina Orduña, Amparo Martinez-Blasco, Javier Sola-Vera, Pedro Moya, Miguel Angel Morcillo, Juan Antonio Ruiz, Rafael Calpena, Francisco-Javier Lacueva

**Affiliations:** Surgery Department, Elche University Hospital, Elche, Spain; Medical Oncology Department, Elche University Hospital and Vega Baja Hospital, Elche, Spain; Pathology Department, Elche University Hospital, Elche, Spain; Gastroenterology Department, Elche University Hospital, Elche, Spain; Surgery Department, Vega Baja Hospital, Elche, Spain; Pathology Department, Vega Baja Hospital, Elche, Spain; Pathology and Surgery Department, Miguel Hernandez University of Elche, Elche, Spain

**Keywords:** Gastric cancer, Perioperative chemotherapy, ECF/X regimen, Surgery, Tumor regression, Survival

## Abstract

**Background:**

We assessed the effectiveness of perioperative MAGIC-style chemotherapy in our series focused on the tumor regression grade and survival rate.

**Methods:**

We conducted a retrospective study of 53 patients following a perioperative regimen of epirubicin, cisplatin, and fluorouracil or capecitabine (ECF/X). Forty-four (83 %) neoplasias were located in the stomach and 9 (17 %) were located at the esophagogastric junction. Perioperative chemotherapy completion, resection, TNM staging, the tumor regression grade (Becker’s classification) and survival were analyzed.

**Results:**

Forty-five patients (85 %) completed the 3 preoperative cycles. R0 resection was achieved in 42 (79 %) patients. Thirty-five (66 %) patients completed the 3 postoperative cycles. Nine carcinomas (17 %) were considered major responders after preoperative chemotherapy. With multivariate analysis, only completion of perioperative chemotherapy (HR: 0.25; 95%CI: 0.08 – 0.79; p = 0.019) was identified as an independent prognostic factor for disease-specific survival. However, the protective effect of perioperative therapy was lost in patients with ypT3-4 and more than 4 positive lymph nodes (HR: 1.16; 95%CI: 1.02 – 1.32; p = 0.029). The tumor regression grade (major vs minor responders) was at the limit of significance only with univariate analysis. The 5-year overall and disease-specific survival rates were 18 % and 22 % respectively.

**Conclusions:**

The percentage of major responder tumors after preoperative chemotherapy was low.

Completion of perioperative ECF/X chemotherapy may benefit patients with gastric carcinomas that do not invade the subserosa with few positive lymph nodes.

## Background

In Europe, gastric carcinoma patients have an average 5-year survival rate of approximately 30 %, although there is wide variation among and within countries [1]. The prognosis in the absence of peritoneal or distant dissemination continues to be poor when lymph node (LN) invasion exists, even when an R0 surgical resection with extended lymphadenectomy is achieved [2, 3]. The effects of extended D2 lymphadenectomy on disease-free survival have not been well established in Western countries [4, 5]. However, some current clinical guidelines recommend a spleen and pancreas preserving D2 lymphadenectomy [6, 7].

Different strategies have been developed to improve the poor prognosis associated with radical surgical resection of localized gastric cancer. Adjuvant radiochemotherapy, which improved the median overall survival rates in the INT 0116 prospective randomized clinical trial [8] has been questioned in patients with lymph node negative disease and after D2 lymphadenectomies [9, 10]. Adjuvant chemotherapy has been consistently effective after D2 lymphadenectomies in prospective, randomized clinical trials involving only Eastern populations [11, 12]. Thus, perioperative treatment including neoadjuvant chemotherapy (NAC) has emerged as the most attractive approach. In fact, the MAGIC [13] and FNCLCC-FFCD [14] prospective randomized clinical trials have found positive effects of the use of NAC on the curative resection rates, disease-free survival and overall survival.

Nevertheless, little is known about tumor regression after neoadjuvant chemotherapy regimen of epirubicin, cisplatin, and fluorouracil or capecitabine (ECF/X), although partial or minimal regression has been reported in 54 % of gastric carcinomas [15]. Assessment of the histopathological changes after NAC is the best method for evaluating tumor response to chemotherapy [16–18] and recent data have shown that complete or subtotal tumor regressions are the only independent prognostic factors for survival [19].

In this study, we assessed the effectiveness of the MAGIC perioperative chemotherapy regimen by analyzing the tumor regression grade, and survival rate.

## Patients and methods

From March 2006 to December 2012 patients with stages II and III gastric and esophagogastric junction (EGJ) carcinomas underwent a perioperative MAGIC regimen chemotherapy at the Elche University Hospital and the Vega Baja Hospital. Patients showing symptoms of outlet obstruction or morbidity that precluded chemotherapy were excluded. The diagnostic and staging workup included an upper endoscopy with biopsy and a CT scan for all patients. After 2008, EUS was usually performed unless carcinomatosis or distant metastases were suspected based on CT scan. Staging laparoscopy was mostly indicated when CT or EUS showed signs of linitis, tumor invasión of adjacent structures or ascitis. Written informed consent was obtained from all patients.

This study was approved by the ethics committee of the University Hospital of Elche.

The following clinical variables were analyzed in this study: age, gender, location, completion and morbidity after preoperative chemotherapy, type of gastrectomy or esophagectomy, extent of LN dissection as stated by the surgeon, surgical morbidity and mortality, completion and morbidity of postoperative chemotherapy, recurrence, and overall and specific survival. The histopathological variables analyzed were the depth of invasion (ypT), total and positive LNs retrieved, LN staging (ypN), carcinomatosis, peritoneal lavage cytology, liver metastasis (ypM), histological type, grade, and tumor regression grade according to Becker’s classification [17]. Tumors with complete regression (grade 1a) or subtotal (<10 % of residual tumor) regression (grade 1b) were considered major responders. Tumors with partial (10–50 % residual tumor) regression (grade 2), or with minimal or no regression (>50 % residual tumor) (grade 3) were classified as minor responders. Gastric and EGJ tumors were staged according to the 7^th^ edition International Union Against Cancer (UICC) TNM pathological classification.

### Treatment and follow-up

ECF/X regimen chemotherapy was administered for 3 cycles preoperatively and three cycles postoperatively. Each 3-week cycle consisted of epirubicin (E) (50 mg per square meter of body-surface area) by intravenous bolus on day 1, cisplatin (C) (60 mg per square meter) intravenously with hydration on day 1, and fluorouracil (F) (200 mg per square meter) daily for 21 days by continuous intravenous infusion or capecitabine (X) twice a day (625 mg per square meter) for 21 days [20].

After preoperative chemotherapy subtotal or total gastrectomy was undertaken in gastric or type III esophagogastric junction (EGJ) carcinomas. In patients with type I or II EGJ carcinomas, an Ivor Lewis partial esophagectomy was performed. Perigastric lymph node stations (1 to 6) were removed in D1 lymphadenectomies. Additionally, lymph node stations 7, 8a, 9, and 11p were removed in modified D2 lymphadenectomies. Inferior periesophageal and subcarinal lymph nodes were also removed in EGJ type I or II carcinomas. D1 or modified D2 lymphadenectomies were chosen according to the surgeon’s criteria.

Routine follow-up examinations consisted of a clinical interview and blood analyses including tumor markers CEA, CA 19.9 and CA125 every 3 months for the first 2 years after surgery and every 6 months thereafter. CT scans were performed every 6 months the first 2 years after surgery and yearly thereafter.

### Statistical analysis

A Kaplan Meier survival analysis was performed using a log-rank test to estimate differences in the 5-year disease-specific survival rates. Multivariate survival analysis was performed using a Cox regression model. Prognostic factors were compared using hazard ratios with a 95 % confidence interval. To identify independent risk factors and to control for possible bias from patients diagnosed as M1 over the course of perioperative treatment, a Cox regression analysis (stratified by ypM) was performed. The model included variables identified by univariate survival analysis as more influential. SPSS for Windows version 20 was used for the statistical analysis.

## Results

Fifty-three patients were included in the study. The clinical characteristics of the patients are summarized in Table 1. Sixteen patients (30.2 %) were older than 70 years of age. EUS was performed in 32 (60.4 %) patients and staging laparoscopy in 8 (15.1 %) patients, with 6 laparoscopies occurring after EUS. Neoplasias were located in the stomach in 44 (83 %) cases and at the esophagogastric junction in 9 (17 %) cases.

**Table 1:** Demographics of the series

Patients	N = 53
Age	64 (38–78)
Sex	
Male	36 (67.9 %)
Female	17 (32.1 %)
Tumor location	
EG Junction	9 (17 %)
Upper/Middle third	20 (37.7 %)
Distal	22 (41.5 %)
Linitis	2 (3.8 %)
Three preoperative cycles completed	45 (84.9 %)
Three postperative cycles completed	35 (66 %)
Resection	
R0	42 (79.2 %)
Unresected or Paliative	11 (20.8 %)
Lauren’s histological type	
Intestinal	22 (59.5 %)
Diffuse/Signet ring cell	15 (40.5 %)
Grade	
Well or moderately differentiated	14 (37.8 %)
Poorly differentiated or undifferentiated	23 (62.2 %)
ypT	
T0-2	16 (33.3 %)
T3-4	32 (66.7 %)
ypN	
N0	17 (35.4 %)
N1	8 (16.7 %)
N2-3	23 (47.9 %)
ypM	
M0	42 (80.8 %)
M1	10 (19.2 %)
Lymph nodes analyzed	
< 15	25 (52.1 %)
≥ 16	23 (47.9 %)
Tumor regression grading	
1a-b	9 (17.3 %)
2–3	43 (82.7 %)
Recurrence #	
No	16 (38.1 %)
Yes	26 (61.9 %)

Recurrence #: In R0 and M0 patients

### Perioperative chemotherapy

Forty-five (84.9 %) patients completed the 3 preoperative cycles. Ten (18.9 %) patients developed grade 3 or 4 hematologic toxicity, which delayed the administration of the next chemotherapy cycle in 6 cases and caused early termination of chemotherapy in 2 (3.8 %) patients. One patient died from a pulmonary embolism. Ten (18.9 %) patients developed grade 3 or 4 non-hematologic toxicity, which forced an end to chemotherapy in 7 (13.2 %) cases and delayed administration of the next chemotherapy cycle in 2 patients.

Thirty-five (66 %) patients received the 3 postoperative cycles, and thus, they completed all the perioperative treatment. Eleven of those patients developed hematologic toxicity that caused only a delay in the administration of the next cycle. One patient developed grade 3 or 4 non-hematologic toxicity that caused a delay of the next cycle. Nine (17 %) patients were shifted to another chemotherapy regimen, and 2 (3.8 %) patients started a radiochemotherapy Macdonalds’s schema. Reasons for changing the chemotherapy regimen were evidence of residual or advanced disease after surgery in 6 patients, toxicity in 2 patients, and patient’s preference in one case. In case of advanced or residual disease, all patients were shifted to docetaxel, cisplatin and fluorouracil (DCF regimen). Seven (13.2 %) patients did not undergo postoperative treatment.

### Surgery

Peritoneal carcinomatosis, pancreatic infiltration or liver metastasis at the time of surgery precluded resection in 4 (7.5 %) patients. Resection was accomplished in 48 patients (90.6 %). Six partial esophagectomies, 9 subtotal gastrectomies and 33 total gastrectomies were performed. An R0 resection was initially obtained in 44 patients, but positive peritoneal lavage cytology was identified postoperatively in 2 of these patients (ypM1); therefore R0 resection was ultimately achieved in 42 (79.2 %) patients. R1 resection was obtained in 1 patient, with a positive proximal margin and several localized peritoneal nodules on the pancreatic surface. R2 resection was performed in 3 patients; one received only a single cycle of preoperative chemotherapy and also showed several localized peritoneal nodules on the pancreatic surface. The laparotomy findings of ypM1 patients are detailed in Table 2.

**Table 2:** Staging procedures and pathologic findings in M1 patients

Patient	USE (uTN)	Staging laparoscopy	Resection	Pathology findings
#2	np	No	R0	ypT4aN2. TR 3. PLC+
#6	np	No	no	Carcinomatosis.
#7	uT4aN0	No	R2	ypT3aN3b. TR 3. Several + nodules on pancreatic surface.
#11	np	No	R2	ypT3aN3a. TR 3. Several + nodules on pancreatic surface.
#14	np	Yes. No findings	R1	ypT4aN3a.TR 3. Margin +. Several + nodules on pancreatic surface.
#31	np	No	R0	ypT4aN3a. TR 3. PLC+
#35	uT4aN2	No	no	Liver metastasis
#38	uT4aN0	No	no	Pancreatic infiltration. Several + nodules on mesocolon. PLC+
#49	uT4aN0	No	R2	ypT4aN3a.TR 3. Carcinomatosis.
#51	uT4aN1	Yes after NAC: Pancreatic infiltration. Liver metastasis	no	PLC+

USE: Ecoendoscopy. uT1: mucosa and submucosa, uT2: muscular, uT3: subserosa, uT4a: serosa, np: not performed

NAC: Neoadjuvant chemotherapy. PLC: peritoneal lavage cytology

TR: tumor regression grade

No significant correlation was found between extent of the lymph node dissection stated by the surgeon and the number of lymph nodes retrieved by the pathologist. Modified D2 lymphadenectomy was more frequently performed than D1 (38 vs 10 patients), but 16 or more lymph nodes were harvested in only 18 of the 38 (47.4 %). Conversely, at least 16 lymph nodes were harvested in 5 of the 10 (50 %) D1 lymphadenectomies performed. Ten or more lymph nodes were analyzed in 37 (77.1 %) patients.

The morbidity after resection was 20.8 %. The surgical mortality was 0 %.

### Pathological findings

The pathological characteristics of the tumors are detailed in Table 1. Twenty-four (50 %) carcinomas were staged as pT4. The median number and range of the retrieved lymph nodes was 14.5 (5-43). Thirty-one (64.6 %) of the resected specimens had positive lymph nodes.

Nine cases (17 %) were considered major responders after preoperative chemotherapy (2 with complete and 7 with subtotal tumor regression) (Table 3). In addition, 2 patients showed complete or subtotal tumor regression but also had positive lymph nodes and they were classified as minor responders. In 31 (64.6 %) patients minimal or no tumor regression was observed (Tables 2 and 4). Peritoneal lavage cytology was performed in 36 (69 %) patients and was positive in 4 (11.1 %).

**Table 3:** Patients with R0 resection and major response to chemotherapy

Case	LOCATION	USE (uTN)	ypTN	TR	Recurrence
#3	Gastric (A)	np	pT0N0	1a	Death at 5^th^ month due to subdural hematoma
#8	GEJ SWT-I	uT3N1	pT2N0	1b	No
#15	Gastric (C)	uT2N1	pT1b	1b	No
#16	GEJ SWT-II	uT4aN0	pT2N0	1b	Local. Peritoneal
#25	Gastric (F)	uT2N1	pT1bN0	1b	Pleura. Peritoneal
#26	Gastric (C)	uT4aN1	pT0N0	1a	No
#27	Gastric (C)	uT4aN1	pT2N0	1b	No
#37	Gastric (A)	uT4aN1	pT4aN0	1b	Death at 9^th^ month due to cardiopathy
#45	Gastric (A)	np	pT4aN0	1b	Regional. Peritoneal

(A): Antrum, (C): Corpus, (F): Fundus

USE: Ecoendoscopy. uT1: mucosa and submucosa, uT2: muscular, uT3: subserosa, uT4a: serosa, np: not performed

TR: tumor regression, 1a: complete, 1b: subtotal

**Table 4:** Patients with R0 resection and partial or minor response to chemotherapy

Case	Location	Use (uTN)	ypTNM	TR	Recurrence
#1	Gastric (F)	np	T1bN1	3	Local regional. Brain
#4	Gastric (C)	np	T3N3a	3*	Peritoneal
#9	GEJ SWT-I	np	T2N0	2	Local regional
#10	Gastric (C)	np	T4aN0	3	Local regional
#12	GEJ SWT-III	np	T4bN3b	3	Liver. Adrenal
#13	Gastric (C)	uT4aN0	T3N0	3	No
#17	Gastric (C)	np	T4aN0	3	No
#18	Gastric (L)	uT4aN1	T0N3b	1a*	Pleura
#19	Gastric (F)	uT2N1	T2N2	3	No
#20	GEJ SWT-II	uT4aN1	T2N1	2	No. Death at 52^th^ month due to renal cancer
#21	Gastric (A)	uTxN1	T4aN2	2	Local regional
#22	Gastric (C)	uT4aN1	T3N2	3	Local regional
#23	Gastric (A)	uT4aN2	T4aN3a	3	Peritoneal
#24	Gastric (C)	np	T3N2	2	Peritoneal. Pleura. Bone
#28	Gastric (A)	uT4aN0	T4aN2	3*	Lung
#29	Gastric (C)	uT4aN0	T4aN0	3*	Liver
#30	Gastric (C)	uT4aN1	T4aN2	3*	Peritoneal
#32	Gastric (A)	uT3N0	T1bN0	3*	No
#33	Gastric (L)	uT3N1	T3N1	2	Peritoneal. Pleura
#34	Gastric (A)	uT4aN2	T2N0	3*	No
#36	Gastric (A)	uT4aN2	T4aN2	3	Liver
#39	Gastric (A)	uT3N1	T2N1	3	No
#40	Gastric (A)	np	T4aN0	3*	No
#41	GEJ SWT-II	np	T4aN1	3	No
#42	Gastric (A)	uT4aN1	T3N3a	3	Bone
#43	GEJ SWT-II	uT4aN1	T4bN1	3	Local regional
#44	Gastric (A)	uT4aN1	T4aN3a	3	Local regional
#46	Gastric (A)	uT2N1	T1bN1	1b*	Liver. Lung
#47	Gastric (C)	np	T4aN3a	3*	Local regional
#48	Gastric (C)	np	T4aN1	3	Lung
#50	Gastric (A)	np	T4aN2	3	Peritoneal
#52	GEJ SWT-III	np	T4bN3	3	Peritoneal
#53	Gastric (C)	uT4aN1	T4aN2	2	No

(A): Antrum, (C): Corpus, (F): Fundus, (L): linitis

USE: Ecoendoscopy. uT1: mucosa and submucosa, uT2: muscular, uT3: subserosa, uT4a: serosa, np: not performed

TR: tumor regression, 2: partial, 3: minimal, 3*: no regression, 1a* and 1b*: complete or subtotal tumor regression but positive lymph nodes

The major and minor tumor responders are detailed in Tables 3 and 4. Among patients with R0 resection who were classified as minor responders to chemotherapy, 4 tumors were classified as stage IA (1 patient) and stage IB (3 patients) after the pathological study but 2 of them were classified as stage IIA and IIIB by EUS before the treatment’s onset. Five of the 23 (21.7 %) patients staged as uT4 presented with M1 disease at surgery.

### Survival analysis

**Fig. 1:**
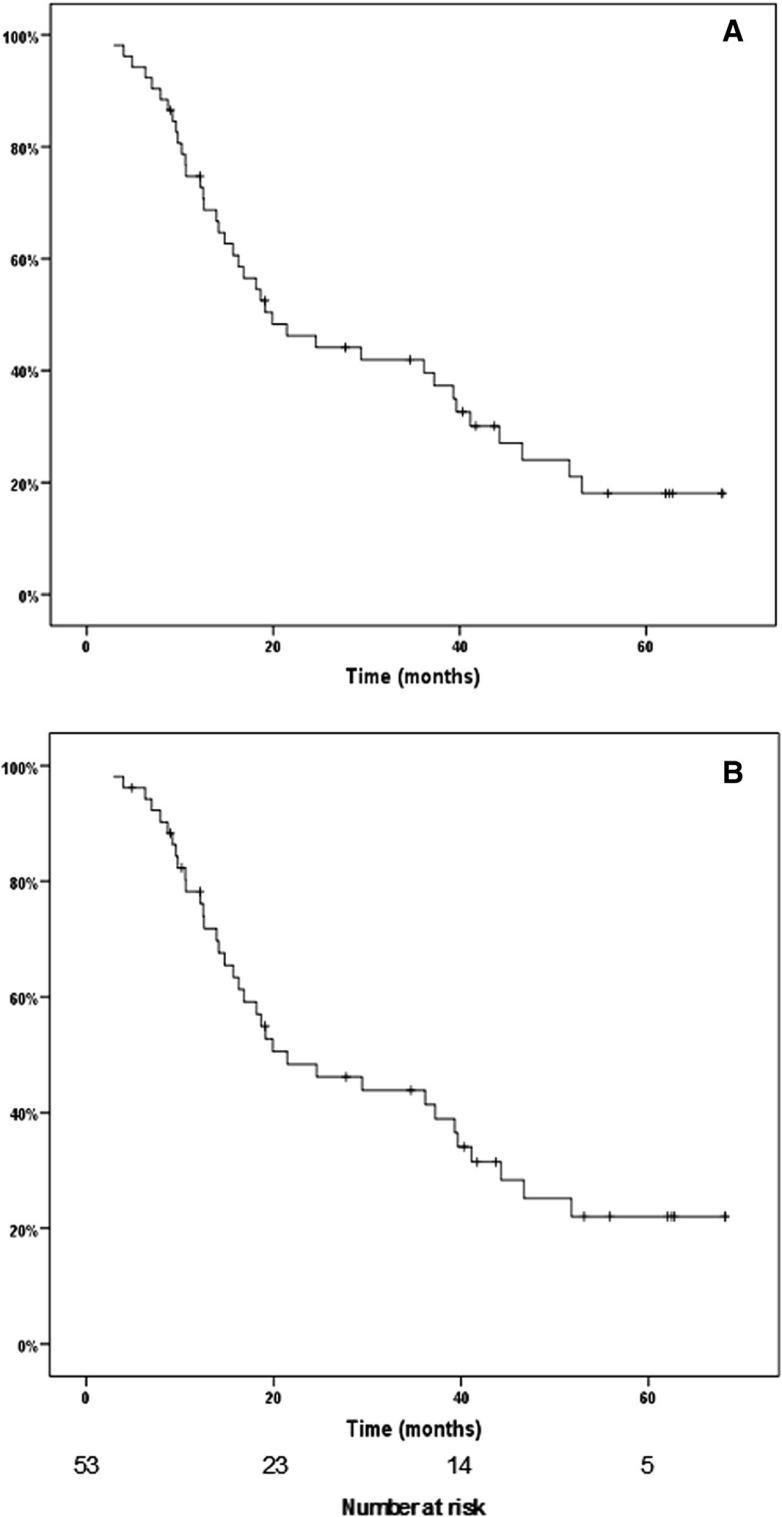
Kaplan-Meier (**a**) overall survival and (**b**) disease-specific survival of the series

The median follow-up of the series was 19 (3–68) months and the median follow-up of the patients alive was 43 (9–68) months. Seventy-nine percent of patients died or were followed for more than 2 years. The 5-year overall and disease-specific survival rates were 18 % and 22 % respectively (Fig. 1a and b).

**Fig. 2:**
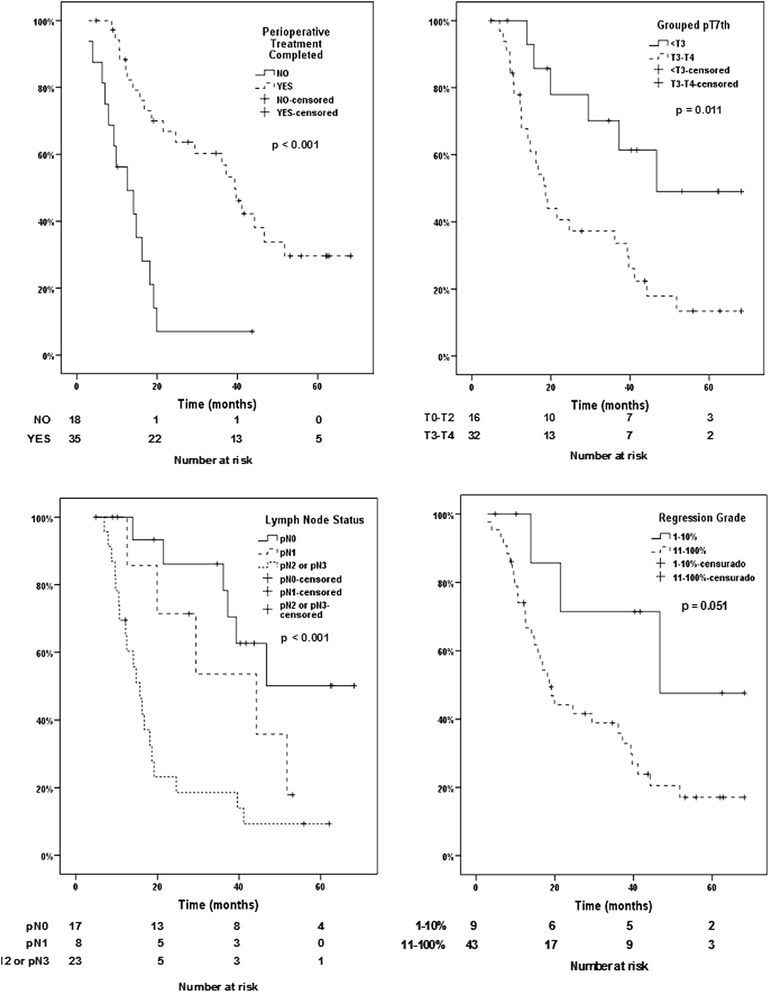
Disease-specific survival of patients according to completion of perioperative chemotherapy, the lymph node status, depth of invasion and tumor regression grade (major and minor responders)

After univariate analysis of the 5-year disease-specific survival, the completion of perioperative chemotherapy (p < 0.001), completion of NAC (p < 0.007), depth of invasion (ypT0-2 vs ypT3-4) (p < 0.01), LN staging (ypN0 vs. ypN2-3) (p < 0.001), number of positive LNs (p < 0.001), and metastasis (ypM0 vs. ypM1) (p < 0.001) were shown to be significant (Fig. 2a–c). LN staging (ypN1 vs. ypN2-3) (p < 0.051) and tumor regression grade (major vs. minor responders) (p < 0.051) were at the limit of significance (Fig. 2c, 2d).

Of the 9 patients with complete or subtotal tumor regression (Becker’s grade 1a and 1b), 3 patients died due to recurrence (33.3 %), 4 patients were disease-free after 3 or more years of follow-up (44.4 %), and 2 patients died during early follow-up due to pulmonary thromboembolism (5^th^ month) and cardiomyopathy (9^th^ month). Of the 33 patients in which R0 resection was achieved but partial, minimal or no tumor regression (Becker’s grade 2 and 3) was observed, 23 (69.7 %) had recurrence, 10 (30.3 %) were disease-free, and 1 patient died due renal cancer progression. The 5-year disease-specific survival rate of patients with regressions grades 1a and 1b was 47.6 %, and was 16.8 % for patients with grades 2 and 3 (Fig. 2d).

A multivariate survival analysis stratified by ypM was performed including the following variables: completed vs. incompleted perioperative chemotherapy, number of positive LNs, ypT3-4 vs. ypT < 3, major vs. minor tumor regression grade, and an interaction factor composed of the completion of perioperative chemotherapy, ypT, and the number of positive LNs (Table 5). Only completion of perioperative chemotherapy (HR: 0.25; 95 % CI: 0.08–0.79; p = 0.019) was identified as an independent prognostic factor for the 5-year disease-specific survival. However, the completion of perioperative chemotherapy, depth of invasion and number of positive LNs were related, and patients with ypT3-4 and more than 4 positive LNs did not show increased survival rates even if they completed perioperative chemotherapy (HR: 1.16; 95 % CI: 1.02–1.32; p = 0.029). The 5-year disease-specific survival rate of the patients who completed perioperative chemotherapy was 29.6 % (Fig. 2a).

**Table 5:** Multivariate survival analysis stratified by ypM

	HR (CI 95 %)	*P* value
Completed perioperative treatment (yes/no)	0.25 (0.08–0.79)	0.019
Number of positive lymph nodes	1.02 (0.93–1.13)	0.653
ypT3-4 vs ypT1-2	1.34 (0.42–4.30)	0.622
Major vs minor regression grade after NAC	0.91 (0.21–3.94)	0.900
Interaction factor: Completed perioperative treatment • ypT3-4 • ypN > 4	1.16 (1.02–1.32)	0.029

Interaction factor: Patients that completed perioperative treatment with ypT3-4 and more than 4 positive lymph nodes

HR: Hazard Ratio

CI: Confidence interval

NAC: Neoadjuvant chemotherapy

## Discussion

In this study, a high percentage of patients completed preoperative chemotherapy (85 %), and a high percentage of R0 resection was achieved (79 %). Completion of preoperative chemotherapy was higher in the perioperative-chemotherapy arm of the MAGIC trial (91 %), but R0 resection was achieved in only 69 % of the cases. Additionally, a higher percentage of our patients completed all 3 postoperative cycles (66 %) compared to those in the MAGIC trial (42 %) [13]. In our study, one patient died during the neoadjuvant period due to pulmonary embolism. It has been reported that up to 3 % of lethal thrombo-embolic episodes occur during the preoperative period [15].

Completion of perioperative chemotherapy was the only independent prognostic factor in our study, and it was associated with a significant improvement of the 5-year specific-survival rate of these patients. However, the beneficial effects of perioperative chemotherapy were not observed in patients that had both tumors infiltrating subserosa or beyond and who had more than 4 positive lymph nodes. These data suggest that the impact of the ECF/X regimen on survival is mild. The overall and 5-year disease-specific survival rate of the series was quite poor compared to the 36 % obtained in the perioperative-chemotherapy group of the MAGIC trial, despite the higher percentage of R0 resection achieved in our study. This difference cannot be easily explained by different pathological findings, because the number of patients with tumors reaching the serosa or beyond (50 %), and tumors without lymph node metastasis (35 %) were very similar in this study and the MAGIC trial (48 % and 31 % respectively). Nevertheless, the higher percentage of pN3 tumors found in our study (27 % vs. 16 %) may lead to poorer 5-year survival rates. Finally, a recent study [21] reported a similar overall survival to that achieved in the MAGIC trial but also the percentage of pN2-pN3 was significant lower (29 %) than the shown in our study (48 %).

Little information is available on the tumor regression grade produced by the preoperative ECF/X regimen in gastric cancer [15, 22]. These reported studies used different classification systems to assess tumor regression, which prevents direct comparisons with our findings. In our study, complete or subtotal regression was only observed in 17 % of carcinomas, but these patients had better 5-year survival rates compared to those with partial or minor tumor regression. This relatively low percentage of major responders has been observed in other gastric cancer studies in which different chemotherapy regimens or radiochemotherapy schemas were preoperatively administered [19, 23, 24]. Notably, patients with subtotal or even complete tumor regression of the primary tumor may also have LN metastasis which occurred in 2 of the patients in this study, a finding that has previously been reported by others [25]. Major tumor regression was consistently supported comparing the uTN to ypTN classifications in this group of carcinomas. Several studies agree that complete or subtotal regression, but not partial regression, is associated with higher disease-free survival rates [19, 24, 25]. In a large series analyzing tumor regression after different neoadjuvant cisplatin-based regimens [19], multivariate analysis identified only complete or subtotal tumor regression and ypN status as independent prognostic factors for survival. Additionally, no difference in the mean survival was identified between patients with partial and minor tumor regression, which is why we combined these patients into one cohort. It must be highlighted that tumor regression was minimal or absent in a large percentage of our patients (67 %). In a similar way, another study of perioperative ECF/X chemotherapy reported less than 50 % tumor regression in 54 % of the cases [15]. This fact suggests that patients with minor or no regression tumors may be at a risk of developing micrometastasis or peritoneal seeding during the neoadjuvant period. In a study of locally advanced gastric carcinomas in which a second laparoscopy was performed after neoadjuvant chemotherapy, free peritoneal tumor cells were detected in 24 % of patients with a previous negative staging laparoscopy, which confirms that the risk is real [26].

It is crucial to identify new biomarkers that predict tumor response or resistance to chemotherapy regimens to avoid the delay of potentially curative surgery in patients with non-responsive tumors [27, 28]. HER2 overexpression showed to be a useful biomarker in inoperable or metastatic gastric and esophagogastric carcinomas for selective treatment with trastuzumab in combination with chemotherapy [29]. On the other hand, ERCC1 nuclear protein expression in clinicopathological studies [30] and leptin expression in *in vitro* studies [31] have been associated with poor pathologic response to platinum-based chemotherapy regimens. Therefore, biomarkers of drugs resistance should be included in future clinical trials.

EUS was useful in selecting the patients included in the perioperative MAGIC regimen except in 2 cases. EUS is recommended for workup staging in some clinical guidelines [6, 7], and it is reportedly more accurate in staging T3 and T4 tumors but less reliable for N staging [32]. However, EUS was not useful in appropriately selecting the uT4a tumors that should undergo a staging laparoscopy for detection of carcinomatosis. Thus, 22 % of the uT4a tumors were staged as upM1 after surgery, a result that is similar to the findings reported by other authors [33].

The most important limitation of this study was the relative small sample size. Other studies confirm how difficult it can be to recruit gastric cancer patients for perioperative chemotherapy, which was evident in the two largest studies with patients from 50 and 28 participant centers that required 8 years to close [13, 14]. Another study carried out in one institution assessed the perioperative MAGIC regimen in one hundred gastro-esophageal carcinomas, but only 32 gastric tumors were included [15].

## Conclusion

Completion of perioperative ECF/X chemotherapy was an independent prognostic factor for the 5-year disease-specific survival, although higher survival rates were impaired in patients with tumors that were invading the subserosa or beyond and more than 4 positive lymph nodes. A small proportion of patients with gastric carcinomas showed complete or subtotal tumor regression after perioperative ECF/X chemotherapy.
